# WT1 and ACE mRNAs of blood extracellular vesicle as biomarkers of diabetic nephropathy

**DOI:** 10.1186/s12967-021-02964-6

**Published:** 2021-07-10

**Authors:** Ehsan Hashemi, Hojat Dehghanbanadaki, Alireza Abbasi Baharanchi, Katayoon Forouzanfar, Ardeshir Kakaei, Seyed Mohammad Mohammadi, Saba Zeidi, Farideh Razi

**Affiliations:** 1 Metabolic Disorders Research Center, Endocrinology and Metabolism Molecular-Cellular Sciences Institute, Tehran University of Medical Sciences, Tehran, Iran; 2 Department of Animal Biotechnology, National Institute of Genetic Engineering and Biotechnology, P.O. Box: 14965-16, Tehran, Iran; 3 Diabetes Research Center, Endocrinology and Metabolism Clinical Sciences Institute, Tehran University of Medical Sciences, Tehran, Iran; 4 Endocrinology and Metabolism Research Center, Endocrinology and Metabolism Clinical SciencesInstitute, Tehran University of Medical Sciences, Tehran, Iran

**Keywords:** Diabetic nephropathy, Extracellular vesicle, Biomarker, WT1, ACE, ELMO1

## Abstract

**Background:**

Diabetic nephropathy (DN) has an increasing global prevalence with excessive health expenditure and burden. Exosomal mRNAs regulate intercellular communications and participate in the pathogenesis of various disorders like DN. This study aimed to assess the expression levels of ACE, ELMO1, and WT1 mRNAs in the blood extracellular vesicles (EVs) of DN patients and diabetic patients without nephropathy (DM group) in comparison to healthy controls and investigate their correlations with the severity of DN.

**Methods:**

The performed investigation is a cross-sectional study of 256 participants including 103 DN patients, 100 DM patients, and 53 healthy controls. The quantification of WT1, ACE, and ELMO1 mRNAs in the blood EVs were executed using qRT-PCR. The ROC analysis was performed to determine the diagnostic accuracy of mRNAs.

**Results:**

DN patients had significantly higher expressed WT1 mRNA (1.70-fold change) and lower expressed ACE mRNA (0.55-fold change) in the blood EVs compared to DM patients and controls. ELMO1 mRNA was not expressed in EVs of any groups. A positive correlation between WT1 mRNA level and urine Alb/Cr ratio (r = 0.602, p < 0.001) and a negative correlation between ACE mRNA expression and urine Alb/Cr ratio within DN patients (r = − 0.474, p < 0.001) was identified. The accuracy of WT1 mRNA and 1/ACE mRNA for predicting incipient DN was 0.63 (95% CI 0.55, 0.72) and 0.62 (95% CI 0.54, 0.71), and for predicting overt DN was 0.83 (95% CI 0.74, 0.92) and 0.75 (95% CI 0.66, 0.83), respectively.

**Conclusions:**

WT1 and ACE mRNAs level in blood EVs were predictors for early diagnosis of DN therefore their quantifications might be used to determine the severity of albuminuria and glomerular injuries.

**Supplementary Information:**

The online version contains supplementary material available at 10.1186/s12967-021-02964-6.

## Introduction

Diabetic nephropathy (DN) is a leading cause of end-stage renal failure globally [1] and is a chronic complication of more than 30% of patients with diabetes mellitus [2, 3]. This condition has an increasing global prevalence with excessive health expenditure and burden, mainly due to high cardiovascular mortality [4, 5]. Thus, early detection of DN has become the main concern of public health. Although the increased urinary albumin excretion test (which is defined as more than 30 mg albumin in 24 h urine sample) is currenrly used for early detection of DN, this biomarker couldn’t accurately predict all DN cases since around 25% of diabetic cases develop progressive loss of kidney function without the presence of proteinuria [6–8]. Besides the procedure of choice to diagnose DN is renal biopsy which is an invasive intervention with bleeding complications including hematuria, perirenal hematoma, and massive internal hemorrhage. For these reasons, most cases of DN have been treated with this diagnosis without a renal biopsy [9]. Even the current best therapeutic options for DN do not completely stop a progression to kidney failure. Therefore, we need to understand new underlying mechanisms of DN development to be able to accurately detect and diagnose DN in the early phase and to retard DN progression.

Exosomes are nanosized (40–100 nm) membrane-derived vesicles released into the extracellular space by various cells [10]. Initially, exosomes were considered to be waste products of the cells [11]; however, we now know that exosomes carry bioactive components like proteins, lipids, metabolites, RNA, and DNA from their original cells [12–14] and participate in intercellular interactions and many biological processes [15]. These biovesicles are present in various body fluids including plasma, serum, lymph, urine, and cerebrospinal fluid [16]. Thus study of these biovesicles is possibly a suitable way to find biomarker for early detection, diagnosis, prevention, prognosis, and targeted therapy of various diseases like DN [9, 17–19]. In this instance, WT1 mRNA gene expression in urinary exosome of DN patients showed significant correlation with the reduction of estimated glomerular filtration rate (eGFR) representing renal function. Consequently this mRNA was a candidate as an attractive predictor for early detection of DN [9]. The expression level of other mRNAs like ACE mRNA was also associated with renal failure progression [20]. On the other hand, single nucleotide polymorphisms (SNP) in the ELMO1 gene correlated with DN development and as a result ELMO1 gene had protection effect on renal function and against DN progression [21–24]. In this study, we aim to determine the expression levels of ACE, ELMO1, and WT1 mRNAs in the blood extracellular vesicles (EVs) of DN patients and diabetic patients without nephropathy (DM patients) compared to healthy controls and investigate whether or not the quantifications of these mRNAs are associated with the degree of albuminuria and the severity of glomerular injuries.

## Methods

### Study design and participants

An observational study consisting of three groups including DN patients, DM patients, and healthy controls was performed. Patients and healthy people attending an outpatient clinic for a routine medical check-up or follow-up of diabetes mellitus were recruited. The participants aging between 30 and 75 years old with DN were assigned to group 1, diabetes mellitus without nephropathy to group 2, and healthy controls with neither diabetes mellitus nor nephropathy to group 3. The type 2 diabetic mellitus was defined based on ADA criteria [25] and nephropathy according to the presence of an increased urinary albumin excretion (albumin ≥ 30 mg/day in 24 h urine sample or an albumin-to-creatinine ratio (Alb/Cr ratio) ≥ 30 mg/g in random urine sample) on two or more consecutive samples [26]. The participants aged fewer than 30 or above 75 years old or who had underlying diseases including urinary infection, cardiovascular disease, pregnancy, severe liver failure, and chronic inflammation or had estimated glomerular filtration rate (eGFR) less than 60 mL/min/1.73 m^2^ were excluded because we had a focus on early DN. All patients who fulfilled informed written consent and met inclusion criteria were enrolled. The enrollment started in November 2018 and continued until December 2019.

This human study was under the Helsinki Declaration and the protocol of the study was evaluated and approved by the Ethics Committee of Endocrine & Metabolism Research Institute and Tehran University of Medical Sciences (Approval ID: IR.TUMS.EMRI.REC.1397.012).

### Data collection and laboratory tests

A sample of venous blood (15 mL) after a minimum of 10 h fasting and random urine was obtained from all participants. Half of the whole blood samples were tested for routine biochemical profile and the remaining half were collected in a sterile EDTA tube for RNA extraction. The routine laboratory tests assessed for study subjects were fasting blood glucose (FBS), urea, creatinine, and HbA1c that were evaluated according to the glucose oxidase, urease, Jaffe, and HPLC methods, respectively. Albumin and creatinine levels of random urine were measured by the Immunoturbidimetry and Jaffe methods, respectively. We administered TOSOH G8 system to assess HbA1c level and Roche commercial kit (Roche, Germany), for the rest of the biochemical tests. The estimated glomerular filtration rate (eGFR) was calculated using the Cockcroft–Gault Equation:

$$ {\text{eGFR}} = \frac{{\left( {140 - {\text{age}}} \right) \times {\text{weight}}}}{{{\text{Serum}}\;{\text{creatinine}}\left( {\frac{{{\text{mg}}}}{{{\text{dl}}}}} \right) \times 72}} \times 0.85\;{\text{if}}\;{\text{woman}}.$$

### Preparation and isolation of blood EVs

10 ml of the blood sample in the EDTA tube were centrifuged at 290×*g* for 20 min at 4 °C for plasma isolation. Then plasma was centrifuged at 12,000×*g* for 20 min at 4 °C and the obtained supernatants were ultracentrifuged at 17,000×*g* for 20 min at 4 °C. Later the supernatants were removed and the pellets suspended in sterile phosphate-buffered saline. Subsequently the pellets were ultracentrifuged at 10,000×*g* for 90 min at 4 °C and resuspended in sterile phosphate-buffered saline and stored at − 80 °C until RNA extraction.

### EVs characterization

#### Scanning electron microscopy (SEM)

SEM was utilized to check the morphology and size of isolated EVs. The isolated EVs were fixed with 2.5% glutaraldehyde in PBS for 1 h and then to enhance electrical conductivity, the surface of samples were coated with a gold layer. Finally the samples were analyzed by VEGA TESCAN.

#### Dynamic light scattering (DLS)

The DLS was applied to check the size of the isolated EVs (Malvern zetasizer, Worcestershire, UK). The suspensions of EVs were carefully added to a cuvette. Three scattering measurements for size and density were recorded.

### RNA extraction, cDNA synthesis and quantitative real-time PCR

The total RNA of EVs was isolated by a TRIzol™ Reagent (USA, Cat. Num: 15596026. Germany) based on the manufacturer’s guideline. The extracted RNAs were stored at − 70 °C until analysis. The quality and quantity of RNA were checked by a Nanodrop (Thermo Scientific 2000). Then, 100 ng of extracted RNA was used for cDNA synthesis using RevertAid First Strand cDNA Synthesis Kit (Fermentas, Cat Num: K1622, USA). The expression of ELMO, ACE2 and Wt1 genes were quantified by a Real-Time qPCR System (Applied Biosystems, USA) using SYBR Green PCR Master Mix (Applied Biosystems) and following specific primers:

- ELMO gene: forward: 5′ AGCATCTGGACAGTCAACACGG 3′ and Reverse: 5′ AGGCAGCGCCACAGAAATTTAC 3′,
- ACE2 gene: forward: 5′ AATGGCAGAGTCCCAACAATCG 3′ and Reverse: 5′ TGTCACTTTCTGCAGCCACACC 3′ and;
- Wt1 gene: forward: 5′ CCAGGCCAGGATGTTTCCTAAAC3′ and Reverse: 5′ TCGAAGGTGACCGTGCTGTAAC3′.

The qRT-PCR measurements were carried out three times using cDNA obtained from three independent experiments. The mRNA expression folds changes was calculated based on the following formula:

$$ \Delta {\text{Ct}} = {\text{Ct}}_{{({\text{target}}\;{\text{gene}})}} {-}{\text{Ct}}_{{({\text{House}}\;{\text{keeping}}\;{\text{gene}})}} ; $$

$$ \Delta \Delta {\text{Ct}} = \Delta {\text{Ct}}_{{({\text{Experimental}}\;{\text{sample}})}} {-}\Delta {\text{Ct}}_{{({\text{Normal}}\;{\text{sample}})}} ; $$

$$ {\text{Gene}}\;{\text{expression}}\;{\text{folds}}\;{\text{change}} = 2^{{ - \Delta \Delta }} . $$

### Statistical analysis

SPSS version 19.0 was executed for statistical analyses. One-way analysis of variance (ANOVA) was conducted for comparisons of continuous variables between three groups and Bonferroni’s method was performed for post hoc analyses. Comparisons of nonparametric variables between three groups were conducted with a chi-square test of independence. Spearman’s correlation test was performed to assess the correlations of mRNAs with demographic and biochemical parameters. Besides, multiple regression analysis using the stepwise method was used to determine significant predictor factors for mRNA level. The diagnostic accuracy of mRNAs was assessed using the area under the receiver operating characteristic curves (AUCs). In this these instances, DN patients were divided into incipient DN group defined as patients with microalbuminuria of 30 to 300 mg/day and overt DN group defined as patients with macroalbuminuria of more than 300 mg/day. Then, the receiver operating characteristic (ROC) curve analyses were performed between incipient DN and DM and also between overt DN and DM to determine the diagnostic performance of mRNAs for predicting incipient DN and overt DN, respectively. Youden index (sensitivity + specificity − 1) was used to determine the best cut-off value of mRNAs. If mRNAs had decreased in incipient DN and overt DN groups compared to the DM group, the inverse fold change of mRNAs was used for ROC analysis. Finally, the sensitivity, specificity, positive predictive value (PPV), and negative predictive value (NPV) of mRNAs at their best cut-off value were assessed. If the continuous variables followed the normal distribution, they were presented with mean ± SD and if not, they were presented with robust statistics, median and interquartile range (IQR). The categorical variables were presented with the number and percentage (n/%). Statistical significance was met at a p-value < 0.05 for all analyses.

## Results

### Demographic and clinical characteristics of study participants

In total, 256 participants were included for statistical analyses, including 103 diabetic patients with nephropathy (DN group), 100 diabetic patients without nephropathy (DM group) and 53 healthy controls. There were 139 men and 117 women with a mean age of 59.48 years old. The demographic and clinical characteristics of participants in each group were shown in Table 1. The ANOVA analyses showed no statistically significant difference in BMI and eGFR between the three groups (p = 0.578 and p = 0.674, respectively) and all participants had an average BMI of 28.77 kg/m^2^ and an average eGFR of 90.69 mL/min/1.73m^2^.

**Table 1:** Demographic and clinical characteristics of study participants: diabetic nephropathy (DN) patients, diabetic patients without nephropathy (DM) and healthy controls

Variables	Group			Total (n = 256)	p value
	DN (n = 103)	DM (n = 100)	Control (n = 53)		
Age (years)	62 ± 9	62 ± 8	52 ± 11	59.48 ± 10.21	< 0.001
Sex (M/F) (male, %)	67/36 (65%)	50/50 (50%)	22/31 (41.5%)	139/117 (54.3%)	0.011
Family history (n, %)	21 (20.4%)	42 (42%)	9 (17%)	72 (28.1%)	< 0.001
Body Mass Index (kg/m^2^)	29.55 ± 4.44	28.29 ± 4.86	30.38 ± 6.83	28.77 ± 4.92	0.578
Fasting glucose (mg/dL)	173 ± 86	152 ± 54	99 ± 16	149.2 ± 69.8	< 0.001
HbA1c (%)	8.8 ± 1.9	7.9 ± 1.5	5.8 ± 0.4	7.8 ± 1.8	< 0.001
Urea (mg/dL)	41 ± 18	34 ± 11	28 ± 7	35 ± 14	< 0.001
Creatinine (mg/dL)	1.23 ± 0.44	.93 ± 0.27	1.01 ± 0.16	1.07 ± 0.36	< 0.001
Urine creatinine	115 ± 62	112 ± 61	161 ± 63	123 ± 65	< 0.001
Urine albumin	373.3 ± 480	12.3 ± 13.7	13.2 ± 19.8	158.8 ± 352.8	< 0.001
Urine Alb/Cr	344.0 ± 450.5	11.0 ± 9.8	7.7 ± 10.0	145.3 ± 329.9	< 0.001
eGFR (mL/min/1.73m^2^)	89.99 ± 36.21	92.85 ± 30.48	87.29 ± 29.09	90.69 ± 31.18	0.674

Data are expressed as mean ± SD for continuous variables and as number and percentage (n, %) for categorical variables

The post hoc analyses of clinical parameters showed HbA1C and urea were significantly different in all pairwise comparisons between groups (all p-value were less than 0.05). Also, there were significant differences in pairwise comparisons of serum Cr in the DN group with DM group and control (p < 0.001 for both) but no difference between the DM group and control (p = 0.36). The pairwise comparisons of FBS showed no significant difference between the DN group and DM group (p = 0.057) while healthy controls had significantly lower FBS than other groups. (p < 0.001 for both) There was no significant difference in urine Alb/Cr ratio between the DM group and control (p = 1.000) but as expected, the DN group had higher urine Alb/Cr ratio compared to other groups (p < 0.001 for both).

### EVs characterization

**Fig. 1:**
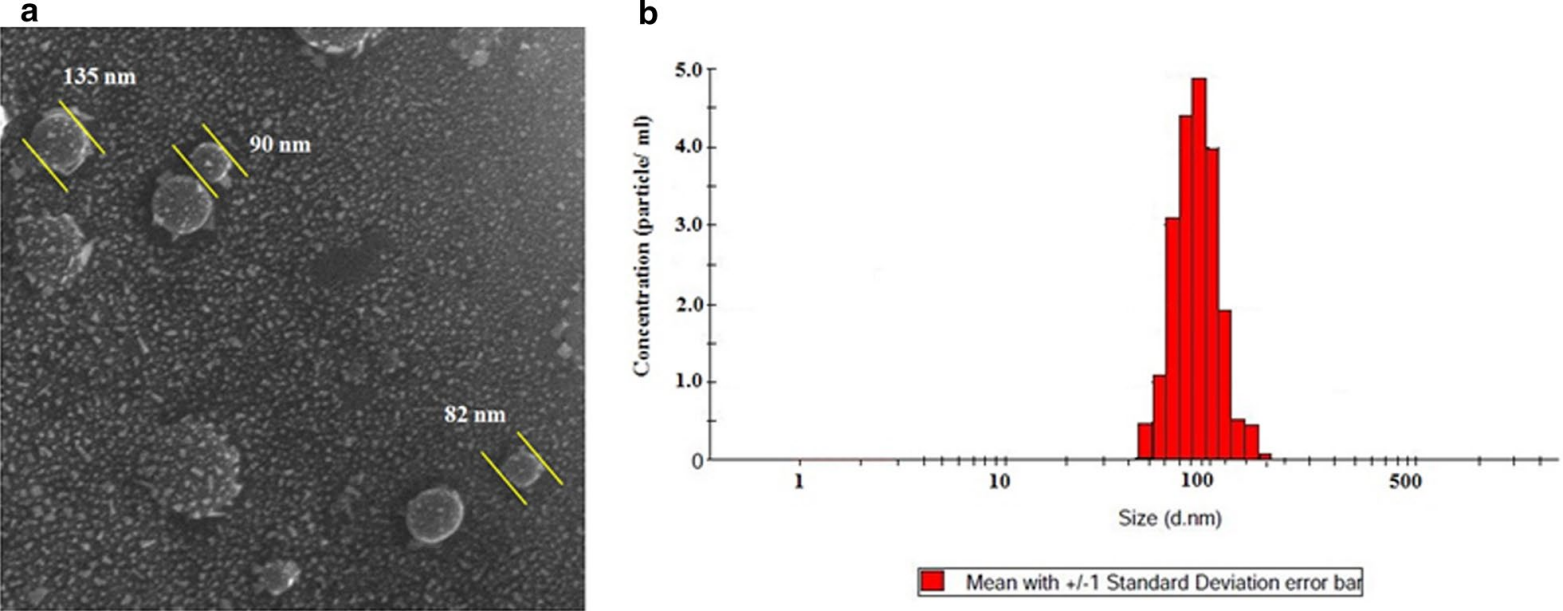
Results of analysis of size and morphology of isolated exosomes by **a** SEM and **b** DLS

As can be seen from Fig. 1 the size and morphology of isolated EVs were analyzed by SEM and DLS. The results showed that the size of isolated EVs was between 80–150 nm (Fig. 1a, b).

### EVs mRNAs profile in three groups

**Fig. 2:**
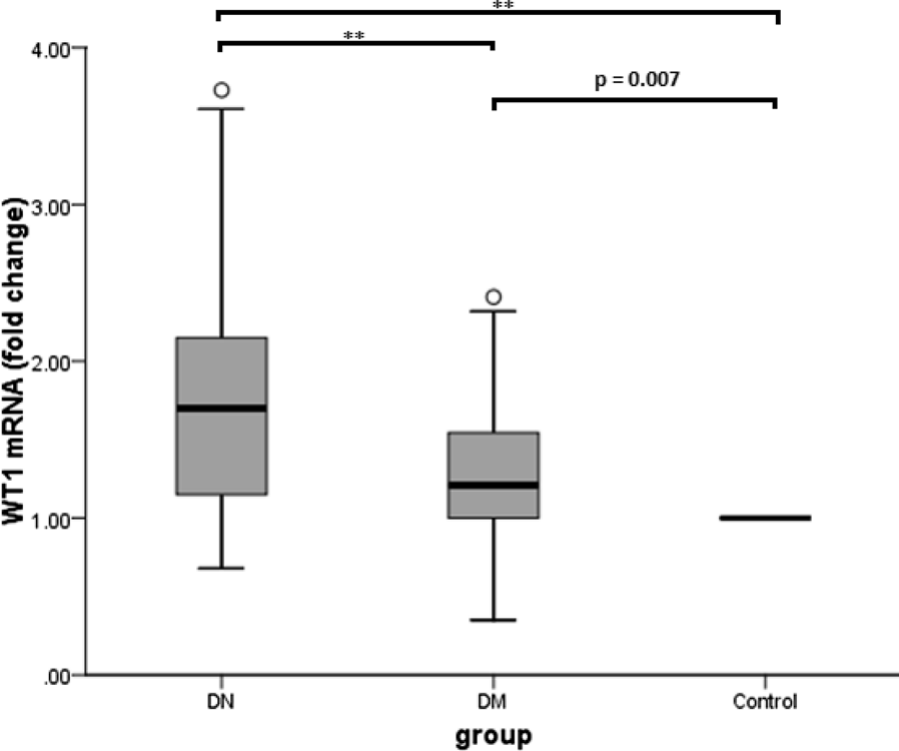
The level of WT1 mRNA in DN patients and DM patients, both compared to the healthy controls. p value is calculated by Post Hoc Bonferroni test of one-way ANOVA. **p < 0.001

After quantitative profiling of mRNA expression, we identified that WT1 mRNA was 1.70-fold up-regulated in the blood EVs of the DN group and 1.21-fold up-regulated in the DM group compared to healthy controls (p < 0.001 and p = 0.007, respectively). The pairwise comparison also showed WT1 mRNA expression in the DN group was significantly higher than the DM group (p < 0.001). Figure 2 shows the fold change and pairwise comparisons of WT1 mRNA expression between groups.

**Fig. 3:**
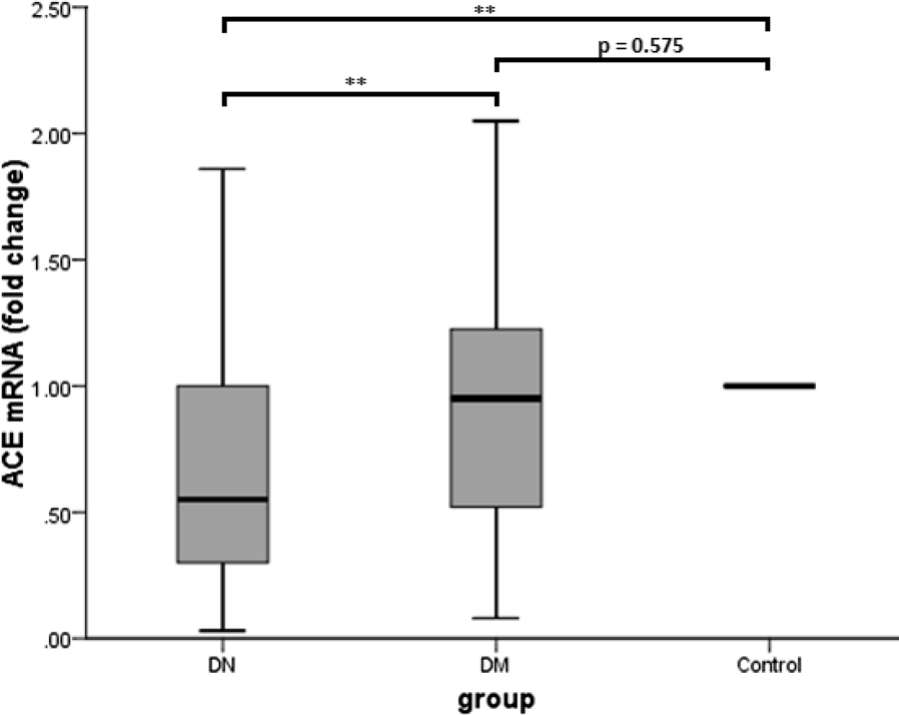
The level of ACE mRNA in DN patients and DM patients, both compared to the healthy controls. p value is calculated by Post Hoc Bonferroni test of one-way ANOVA. **p < 0.001

ACE mRNA was significantly down-regulated in blood EVs of DN patients (0.55-fold change based on healthy controls) compared to diabetic patients and healthy controls (p < 0.001 for both). However, ACE mRNA expression did not significantly differ between diabetic patients and healthy controls (p = 0.575) (Fig. 3).

Additionally, we identified that ELMO1 mRNA was not expressed in significant amounts in the blood EVs of any group. The average expression of WT1, ACE, and ELMO1 mRNAs was shown in Additional file 1: Table S1.

### The association between mRNAs profile and clinical characteristics

Table 2 shows the correlations of WT1 and ACE mRNAs with demographic and clinical parameters of all participants, DN patients, and DM patients, separately. The expression of WT1 mRNA within all categories (total sample, DN group, and DM group) had a positive correlation with levels of HbA1C and FBS (p < 0.01 for all correlation coefficients). The positive correlations were also between WT1 mRNA and urine Alb/Cr ratio in total and in the DN group (r = 0.509, r = 0.609, respectively, p < 0.001 for both). The scatterplot correlation between WT1 mRNA and Alb/Cr ratio within DN patients was shown in Additional file 1: Figure S1. We also observed weak positive correlations of WT1 mRNA with age, urea, and serum Cr in the total sample (p = 0.004, p = 0.002, and p = 0.001, respectively) and with serum Cr in the DN group (p = 0.025).

**Table 2:** Correlations of WT1 mRNA and ACE mRNA with demographic and clinical parameters within total sample, DN group and DM group, separately

	mRNA		Age	BMI	FBS	HbA1c	Urea	S. Cr	eGFR	Alb/Cr
Total	WT1	r	0.222	− 0.167	0.423	0.571	0.193	0.200	0.042	0.509
		p	0.004	0.252	< 0.001	< 0.001	0.002	0.001	0.636	< 0.001
	ACE	r	− 0.101	0.180	− 0.402	− 0.592	− 0.135	− 0.177	0.058	− 0.465
		p	0.190	0.216	< 0.001	< 0.001	0.032	0.005	0.505	< 0.001
DN group	WT1	r	0.161	− 0.207	0.270	0.341	0.020	0.221	− 0.201	0.602
		p	0.239	0.565	0.006	< 0.001	0.845	0.025	0.326	< 0.001
	ACE	r	− 0.110	0.250	− 0.340	− 0.445	− 0.021	− 0.159	0.273	− 0.474
		p	0.424	0.486	0.001	< 0.001	0.836	0.110	0.177	< 0.001
DM group	WT1	r	0.040	− 0.172	0.280	0.518	0.001	0.007	0.138	0.018
		p	0.736	0.331	0.005	< 0.001	0.991	0.944	0.262	0.864
	ACE	r	0.050	0.154	− 0.372	− 0.685	0.064	− 0.025	0.040	− 0.234
		p	0.670	0.386	< 0.001	< 0.001	0.526	0.803	0.747	0.020

*BMI* body mass index, *FBS* fasting blood glucose, *S. Cr* serum creatinine, *eGFR* estimated glomerular filtration rate, *Alb/Cr* urine albumin/creatinine ratio

r = correlation coefficient of spearman, p = p-value

Conversely, the expression level of ACE mRNA in blood EVs was negatively correlated with the levels of HbA1C and FBS within the total sample, DN group, and DM group (p < 0.01 for all correlation coefficients). Also, the ACE mRNA had a significant negative correlation with urine Alb/Cr ratio within the total sample, DN group, and DM group (p < 0.001, p < 0.001 and p = 0.020, respectively). Additional file 1: Figure S2 showed the scatterplot correlation between ACE mRNA and Alb/Cr ratio within DN patients grouped by sex. The level of ACE mRNA also showed negative weak correlations with urea and serum Cr in the total category (p = 0.032 and p = 0.005, respectively).

The multiple linear regression analysis on the factors influencing WT1 mRNA level (age, FBS, HbA1c, urea, serum creatinine, and urine Alb/Cr ratio) showed that HbA1c, serum creatinine, and urine Alb/Cr ratio were the significant predictors for the level of WT1 mRNA (p < 0.001, p = 0.03 and p < 0.001, respectively). Besides, the multiple linear regression analysis on the factors influencing ACE mRNA level (FBS, HbA1c, urea, serum creatinine, and urine Alb/Cr ratio) showed that HbA1c and urine Alb/Cr ratio were the significant predictors for the level of ACE mRNA (p < 0.001 and p = 0.002, respectively) Table 3 shows the results of stepwise multivariate linear regression on the factors influencing WT1 mRNA and ACE mRNA.

**Table 3:** Stepwise multivariate linear regression analyses on the factors influencing WT1 mRNA and ACE mRNA

Predictor factors^a^	Unstandardized coefficients		Standardized coefficients	t	p-value*
	B	SE	Beta		
Dependent variable: WT1 mRNA					
Constant	− 0.024	0.197		− 0.120	0.905
HbA1c	0.142	0.021	0.426	6.661	< 0.001
S. Cr	0.236	0.113	0.130	2.081	0.039
Alb/Cr ratio	0.001	< 0.001	0.322	4.832	< 0.001
Dependent variable: ACE mRNA					
Constant	1.730	0.097		17.829	< 0.001
HbA1c	− 0.113	0.012	− 0.502	− 9.087	< 0.001
Alb/Cr ratio	< 0.001	< 0.001	− 0.176	− 3.190	0.002

*FBS* fasting blood glucose, *S. Cr* serum creatinine, *Alb/Cr ratio* urine albumin/creatinine ratio, *SE* standard error

^a^The following independent variables were adopted for multivariate linear regression analysis of WT1 mRNA: age, FBS, HbA1c, urea, serum creatinine and urine Alb/Cr ratio

*p-value < 0.05 was considered statistically significant

### Accuracy of WT1 mRNA and 1/ACE mRNA for predicting incipient DN

**Fig. 4:**
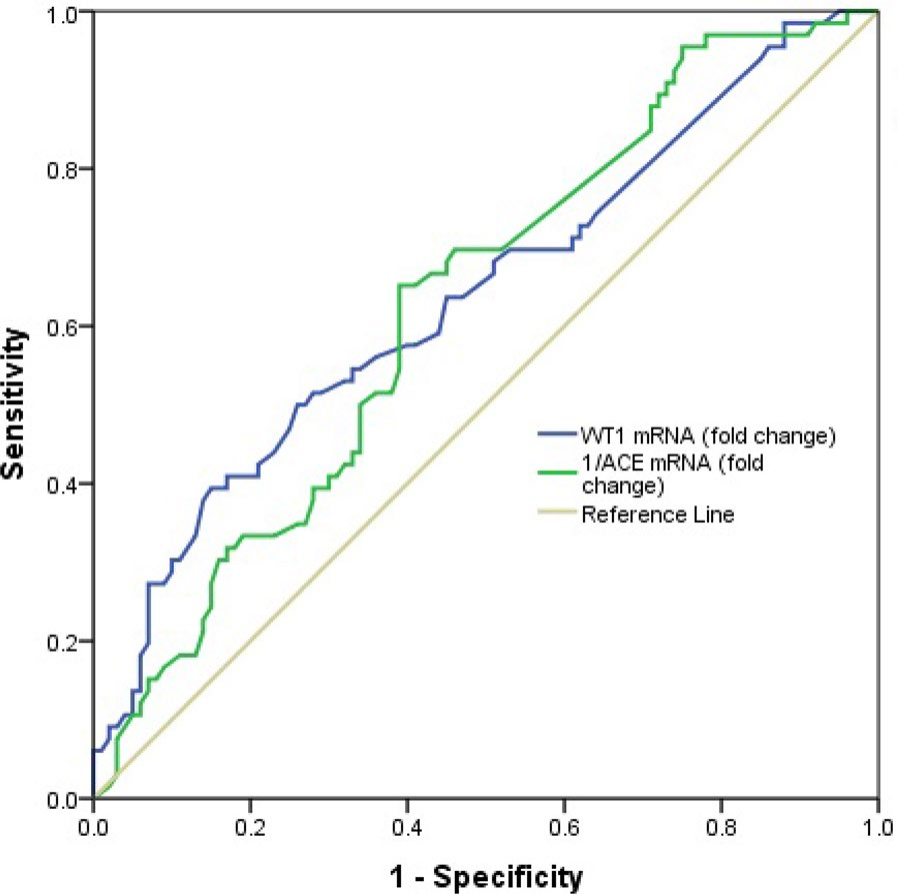
The receiver operating characteristic curves of WT1 mRNA and 1/ACE mRNA for predicting incipient DN

Of 103 DN patients, 66 patients had incipient DN and 37 patients had overt DN. The AUC of WT1 mRNA for predicting incipient DN was 0.63 (95% CI 0.55, 0.72, p = 0.003) (Fig. 4). WT1 mRNA at a cut-off value of 1.50 had a sensitivity of 50%, a specificity of 74%, a PPV of 55.9% and a NPV of 69.1%. Since ACE mRNAs had decreased in incipient DN compared to DM, inverse fold change of ACE mRNA was used for ROC analysis. The AUC of 1/ACE mRNA for predicting incipient DN was 0.62 (95% CI 0.54, 0.71, p = 0.005) (Fig. 4). 1/ACE mRNA at a cut-off value of 1.15 showed a sensitivity of 65.2%, a specificity of 61%, a PPV of 52.4% and a NPV of 72.6%. Table 4 shows the diagnostic performance of WT1 mRNA and 1/ACE mRNA for predicting incipient DN.

**Table 4:** The diagnostic accuracy of WT1 mRNA and 1/ACE mRNA for predicting incipient DN

Parameters	Threshold values	Sensitivity (%)	Specificity (%)	PPV (%)	NPV (%)	AUC (95% CI)	p value
WT1 mRNA	1.50	50.0	74.0	55.9	69.1	0.63 (0.55, 0.72)	0.003
$\frac{1}{{{\text{ACE}}\;{\text{mRNA}}}}$	1.15	65.2	61.0	52.4	72.6	0.62 (0.54, 0.71)	0.005

*AUC* area under the curve, *PPV* positive predictive value, *NPV* negative predictive value

### Accuracy of WT1 mRNA and 1/ACE mRNA for predicting overt DN

**Fig. 5:**
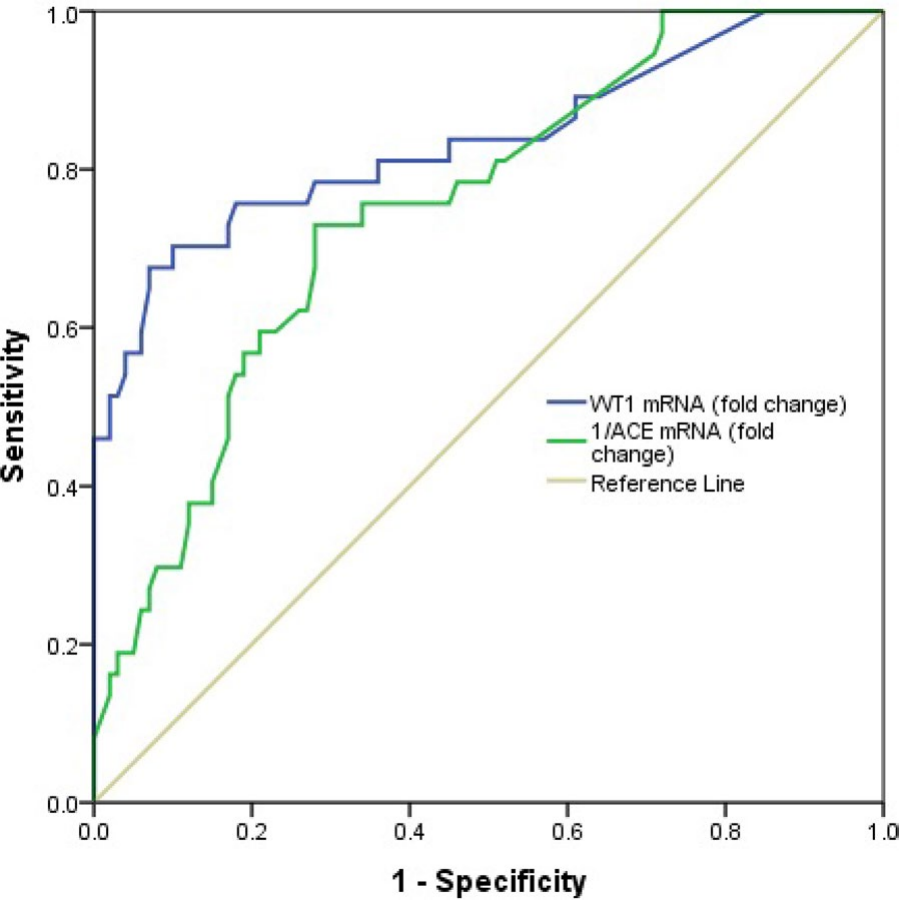
The receiver operating characteristic curves of WT1 mRNA and 1/ACE mRNA for predicting overt DN

The ROC analysis between 37 overt DN patients and 100 DM patients showed that WT1 mRNA had a good accuracy of 0.83 (95% CI 0.74, 0.92, p < 0.001) for predicting over DN (Fig. 5). The sensitivity, specificity, PPV, and NPV of WT1 mRNA at a cut-off value of 1.89 were 67.6%, 93%, 78.1%, and 88.5%, respectively. 1/ACE mRNA was calculated and used in ROC analysis since overall ACE mRNA had also decreased in overt DN compared to DM. Figure 5 shows that 1/ACE mRNA had an accuracy of 0.75 (95% CI 0.66, 0.83, p < 0.001) for predicting overt DN. The sensitivity, specificity, PPV, and NPV of 1/ACE mRNA at a cut-off value of 1.80 were 73%, 72%, 49%, and 87.8%, respectively. Table 5 shows the diagnostic performance of WT1 mRNA and 1/ACE mRNA for predicting overt DN.

**Table 5:** The diagnostic accuracy of WT1 mRNA and 1/ACE mRNA for predicting overt DN

Parameters	Threshold values	Sensitivity (%)	Specificity (%)	PPV (%)	NPV (%)	AUC (95% CI)	p value
WT1 mRNA	1.89	67.6	93.0	78.1	88.5	0.83 (0.74, 0.92)	< 0.001
$\frac{1}{{{\text{ACE}}\;{\text{mRNA}}}}$	1.80	73.0	72.0	49.0	87.8	0.75 (0.66, 0.83)	< 0.001

*AUC* area under the curve, *PPV* positive predictive value, *NPV* negative predictive value

## Discussion

In this study, a quantitative RNA sequencing was used to assess the levels of WT1, ACE, and ELMO1 mRNAs expression in the blood EVs of DN patients. As a result, WT1 mRNA was significantly up-regulated and ACE mRNA was significantly down-regulated in the blood EVs of DN patients compared to DM patients and healthy controls. However, ELMO1 mRNA did not express insignificant amounts to detect in any groups. In addition, WT1 and ACE mRNAs had a significant association with the degree of albuminuria in DN. In this instance, we suggested new pathways in the pathogenesis of nephropathy in type 2 diabetes mellitus and consequently provided novel biomarkers as predictors for early detection, accurate diagnosis, and targeted therapy of DN. Although several previous studies had focused on the profile of mRNAs and microRNAs in the urine exosomes as well as circulating exosomal microRNAs in DN [9, 17, 18, 23–31], to our knowledge so far, this is the first study on the mRNAs specifically in the blood exosomes of DN patients. One of the advantages of blood exosomes over urine exosomes is that the concentration of urine exosomes and subsequently its overall contents were affected by food uptake and urine volume while blood exosomal compositions did not depend on these factors [32].

DN patients had podocyturia due to glomerular dysregulation and the levels of podocyte-specific molecules increased in this state. Abe et al. [9] reported that exosomal WT1 mRNA derived from podocytes was significantly higher in the urine of DN patients compared to patients with minimal change nephrotic syndrome (MCNS) and healthy controls. In addition, their longitudinal study showed that WT1 mRNA level could serve as a predictor for eGFR decline in DN. In our study, eGFR did not significantly differ between the three groups and all participants had a good renal function in spite of the presence of albuminuria. Thus we found no correlation of WT1 and ACE mRNAs with eGFR due to this issue. In addition, since DN patients were in a good state of renal function with normal eGFR, we could assume that DN patients were in the early phase of nephropathy, and blood EVsWT1 and ACE mRNAs might be good biomarkers for diagnosis of early DN.

Alnahal et al. [20] investigated ACE mRNA expression in the urine exosomes of patients with glomerulonephritis (GN) and reported that GN patients had a higher level of ACE mRNA than control group and they suggested the role of the renin–angiotensin system (RAS) in the GN development. However, our findings showed contradictory results that DN patients had a lower level of ACE mRNA gene expression. Since we conducted an observational study, we could not interpret any causal effect from this finding and we required a well-designed experimental trial to identify the role of RAS on DN progression.

We identified that ELMO1 mRNA did not express in significant amounts in any group of study while genomic-wide association studies reported that ELMO1 gene had a strong protective effect on DN development [23].

Finally, a positive correlation between WT1 mRNA expression and Alb/Cr ratio and a negative correlation between ACE mRNA expression and Alb/Cr ratio was found. Thus, quantification of WT1 and ACE mRNAs in blood exosomes reflects the degree of albuminuria and the severity of nephropathy in DN patients, suggesting these mRNAs could serve as predictors of DN progression. As discussed, the role of new biomarkers like EVs and genetic materials of its content [33] and new protein markers like periostin and cyclophilin A [34] in development and diagnosis of DN in early stages is priceless.

There are several limitations in this study. First, this is an observational study with a cross-sectional design and we could not interpret our findings in the causal relationship. Second, several demographic and clinical characteristics of participants differed significantly between three groups and this issue might influence the expression levels of mRNAs. Third, we used a urine sample (the presence of micro/macroalbuminuria) for the detection of nephropathy instead of renal biopsy since most DN participants had classical presentations of nephropathy. Fourth, as we collected samples at a specific time, the lead time bias may have occurred. Finally, the effect of medication was not considered to check any differences for taken medication. Meanwhile, we required a larger multi-center study with different ethnicities and areas to confirm our results.

## Conclusions

In summary, we identified that DN patients had a higher level of WT1 mRNA gene expression and a lower level of ACE mRNA gene expression in the blood EVs. Thus these mRNAs might be used as predictors for the early detection of DN. Furthermore, some DM patients with a high and low level of WT1 and ACE may develop to DN and these markers can be an early predictor of DN. In addition, quantification of these mRNAs was strong predictors for the severity of albuminuria and glomerular injuries. Our findings suggested a new pathway in the pathogenesis of DN development and further studies on these blood EVs mRNAs are warranted to discover novel biomarkers that would be targeted in early detection, diagnosis, prevention, prognosis, and treatment of DN patients.

## Supplementary Information

**Additional file 1: Table S1.** Average fold change of mRNAs in DN patients and DM patients compared to healthy controls. Data are expressed as median (IQR). n.s: not significant. *p-value is calculated by the one-way ANOVA test. **Figure S1.** Correlation between WT1 mRNA and urine Alb/Cr ratio in DN patients grouped by sex. **Figure S2.** Correlation between ACE mRNA and urine Alb/Cr ratio in DN patients grouped by sex.

## Data Availability

The datasets used and/or analyzed during the current study are available from the corresponding author on reasonable request.

## Abbreviations

WT1: Wilms’ tumor gene 1
ACE: Angiotensin-converting enzyme
ELMO1: Engulfment and cell motility 1
DN: Diabetic nephropathy
DM: Diabetic mellitus
EV: Extracellular vesicle
BMI: Body mass index
FBS: Fasting blood glucose
Cr: Creatinine
Alb: Albumin
Alb/Cr ratio: Albumin-to-creatinine ratio
eGFR: Estimated glomerular filtration rate
SNP: Single nucleotide polymorphisms
MCNS: Minimal change nephrotic syndrome
GN: Glomerulonephritis
RAS: Renin–angiotensin system
AUC: Area under the curve
PPV: Positive predictive value
NPV: Negative predictive value
