# Supplementary material for: WT1 and ACE mRNAs of blood extracellular vesicle as biomarkers of diabetic nephropathy
Source: J Transl Med. 2021 Jul 10;19:299. doi: 10.1186/s12967-021-02964-6 (PMC8272332; doi:10.1186/s12967-021-02964-6)
Supplement: Supplementary file 1 — Additional file 1: Table S1. Average fold change of mRNAs in DN patients and DM patients compared to healthy controls. Data are expressed as median (IQR). n.s: not significant. *p-value is calculated by the one-way ANOVA test. Figure S1. Correlation between WT1 mRNA and urine Alb/Cr ratio in DN patients grouped by sex. Figure S2. Correlation between ACE mRNA and urine Alb/Cr ratio in DN patients grouped by sex. [file 12967_2021_2964_MOESM1_ESM.docx]

**Additional file 1**

Table S1 Average fold change of mRNAs in DN patients and DM patients compared to healthy controls

| **mRNAs** | **Fold change** | | **Total P value*** |
| --- | --- | --- | --- |
|  | DN group | DM group |  |
| **WT1** | 1.70(1.14, 2.17) | 1.21 (1, 1.55) | <0.001 |
| **ACE** | 0.55 (0.29, 1.00) | 0.95(0.52, 1.23) | <0.001 |
| **ELMO1** | n.s | n.s | n.s |

Data are expressed as Median (IQR). n.s: not significant

*P value is calculated by One-way ANOVA test.


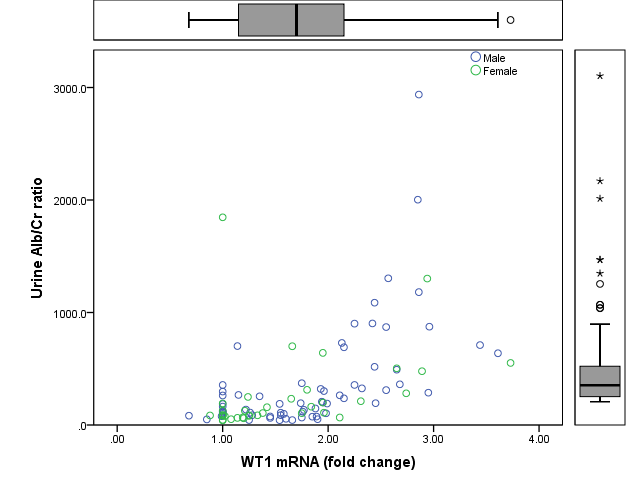


Figure S1. Correlation between WT1 mRNA and urine Alb/Cr ratio in DN patients grouped by sex.


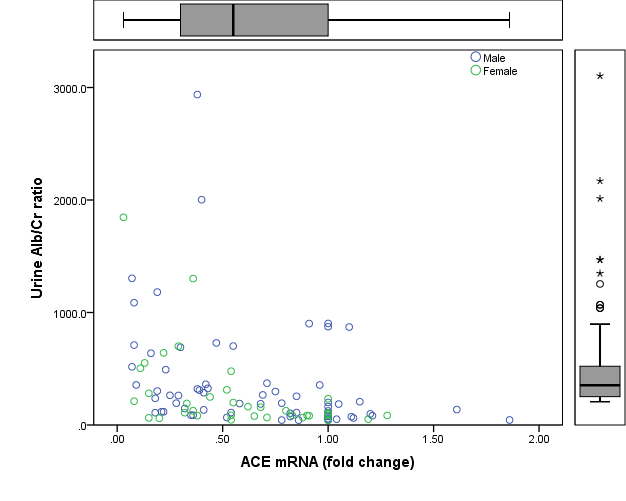


Figure S2. Correlation between ACE mRNA and urine Alb/Cr ratio in DN patients grouped by sex.
